# The Association Between Lower-Limb Muscle Mass Asymmetry and Hip Fracture Laterality in Elderly Women: A Retrospective, Dual-Energy X-ray Absorptiometry (DXA)-Based Study

**DOI:** 10.7759/cureus.102797

**Published:** 2026-02-01

**Authors:** Yonghyun Yoon, Ji Hyo Hwang, Cheol Lee, Hyunjong Yu, Anwar Suhaimi, Teinny Suryadi, King Hei Stanley Lam

**Affiliations:** 1 Department of Orthopedic Surgery, Hallym University Kangnam Sacred Heart Hospital, Seoul, KOR; 2 Department of Orthopedics, IncheonTerminal Orthopedic Surgery Clinic, Incheon, KOR; 3 Department of Anesthesiology and Pain Medicine, Wonkwang University School of Medicine, Iksan, KOR; 4 Rehabilitation Medicine, University Malaya Medical Centre, Kuala Lumpur, MYS; 5 Rehabilitation Medicine, University Malaya, Kuala Lumpur, MYS; 6 Physical Medicine and Rehabilitation, Synergy Clinic, Jakarta, IDN; 7 Department of Physical Medicine and Rehabilitation, Hermina Podomoro Hospital, Jakarta, IDN; 8 Faculty of Medicine, The Chinese University of Hong Kong, Sha Tin, HKG; 9 Faculty of Medicine, The University of Hong Kong, Hong Kong, HKG; 10 The Board of Clinical Research, The Hong Kong Institute of Musculoskeletal Medicine, Kowloon, HKG

**Keywords:** dual-energy x-ray absorptiometry, fracture laterality, hip fracture, lean mass plus bone mineral content, lower-limb asymmetry, muscle mass asymmetry

## Abstract

Background and objective

While sarcopenia is a recognized risk factor for hip fracture, the role of side-to-side lower-limb muscle mass asymmetry in determining fracture laterality remains unclear. This study aimed to investigate whether dual-energy X-ray absorptiometry (DXA)-derived lower-limb muscle mass asymmetry is associated with hip fracture laterality in elderly women.

Methods

We retrospectively analyzed a cohort of 147 women aged ≥65 years with unilateral hip fractures who underwent DXA. Patients were categorized according to fracture side: left (n = 71) or right (n = 76). Lean mass (L), lean mass plus bone mineral content (LB), fat mass, and muscle indices, including the appendicular skeletal muscle index (ASMI) and skeletal muscle index (SMI), were compared between groups. Inter-limb asymmetry was assessed using left-right differences in lower-limb DXA-derived parameters.

Results

The left-fracture group demonstrated significantly higher left-leg lean mass plus bone mineral content compared with the right-fracture group (4928.0 ± 1223.3 g vs. 4539.8 ± 873.8 g, p = 0.030). Analysis of inter-limb differences revealed a significant association between fracture laterality and lower-limb lean mass plus bone mineral content asymmetry (p = 0.004). Overall, 62.6% (92/147) of fractures occurred on the side with greater lower-limb lean mass plus bone mineral content.

Conclusions

In elderly women, hip fractures are more likely to occur on the side with relatively greater lower-limb lean mass plus bone mineral content. These findings suggest that inter-limb muscle mass asymmetry, rather than absolute muscle deficiency alone, may contribute to hip fracture risk, potentially through asymmetric load transmission during falls.

## Introduction

Hip fractures in older adults impose substantial morbidity, mortality, and socioeconomic burdens [1]. Although acute surgical management is well established, effective strategies for primary prevention and the prevention of secondary contralateral fractures remain suboptimal [2]. Established risk factors include osteoporosis, sarcopenia, and elevated FRAX scores, yet optimal risk stratification and targeted prevention strategies are still debated [3]. Clinically, it is often assumed that fractures occur on the “weaker” side, which is presumed to have lower muscle mass. While sarcopenia increases the risk of falls, the contribution of inter-limb musculoskeletal asymmetry to hip fracture laterality has not been specifically investigated [4,5]. Most commonly used muscle and bone assessments focus on absolute values and implicitly assume bilateral symmetry.

Importantly, fracture risk assessment based solely on dual-energy X-ray absorptiometry (DXA) T-scores may be insufficient. Hip-spine bone mineral density (BMD) discordance is common in elderly hip fracture patients, suggesting that site-specific skeletal vulnerability can differ despite similar global measures [6]. In addition, trabecular bone score (TBS) provides complementary information on trabecular microarchitecture independent of BMD, and proximal femoral cortical geometry, such as cortical thickness and cortical thickness index (CTI), has also been associated with hip fracture risk [7,8]. Together, these data indicate that structural characteristics not captured by a single T-score may meaningfully contribute to hip fracture susceptibility.

Building on our prior work emphasizing site-specific bone quality beyond T-scores, we extended this concept to the musculoskeletal domain. We hypothesized that hip fracture laterality may be associated with lower-limb muscle mass asymmetry. Accordingly, this study aimed to investigate the relationship between lower-limb muscle mass asymmetry and hip fracture laterality in older women.

## Materials and methods

Study design and participants

**Figure 1:**
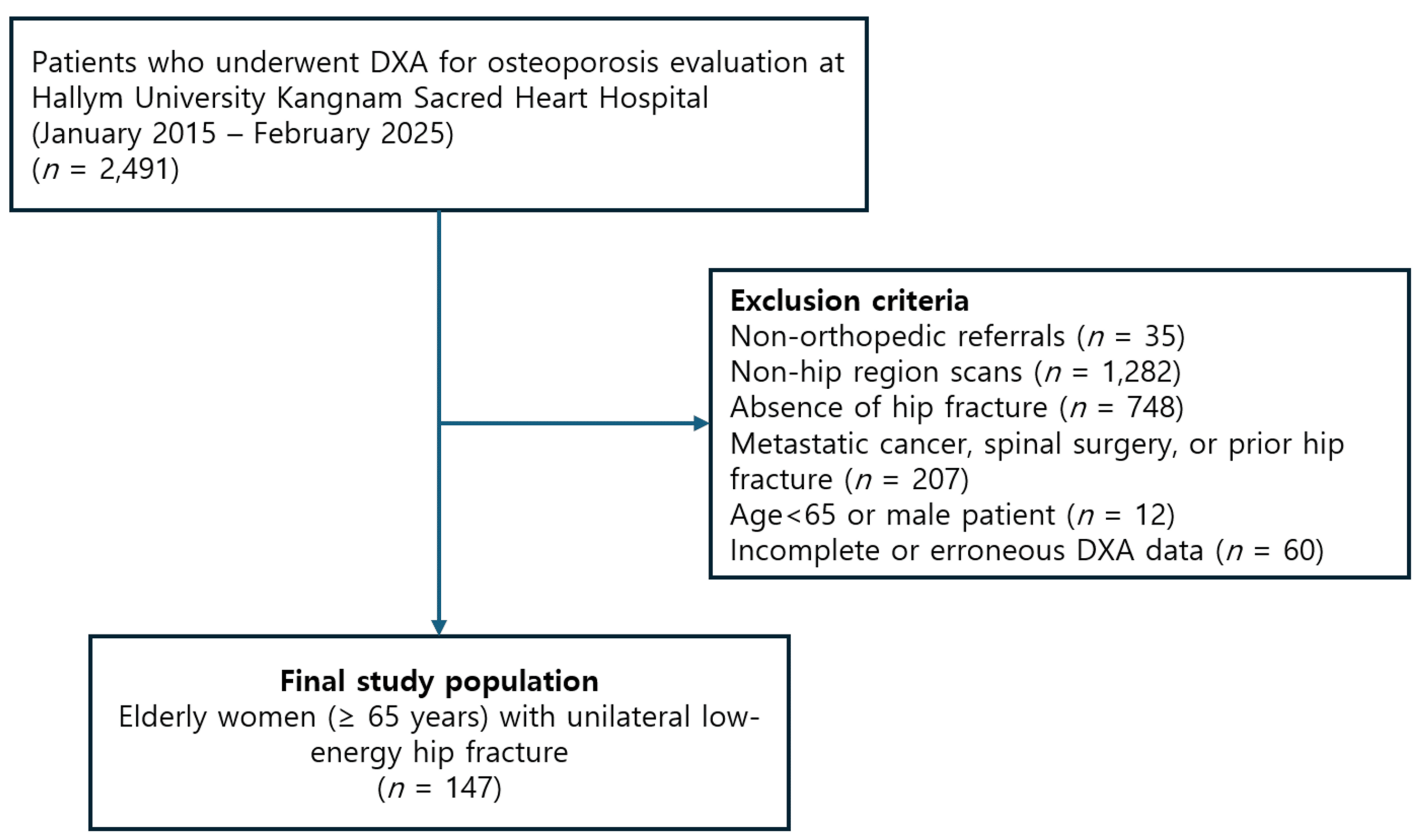
Flowchart illustrating patient selection and exclusion criteria Exclusion categories are shown cumulatively and may not be mutually exclusive DXA: dual-energy X-ray absorptiometry

This retrospective observational study analyzed data from patients who underwent DXA for osteoporosis evaluation at Hallym University Kangnam Sacred Heart Hospital between January 2015 and February 2025. From an initial pool of 2491 patients, we applied the following exclusion criteria: non-orthopedic referrals (n = 35), non-hip region scans (n = 1282), absence of hip fracture (n = 748), history of metastatic cancer, spinal surgery, or prior hip fracture (n = 207), age <65 years or male gender, and incomplete or erroneous DXA data (e.g., missing limb values, scan artifacts, n = 60). The final cohort comprised 147 female patients aged ≥65 years with a unilateral, low-energy hip fracture. The study was approved by the Institutional Review Board of Hallym University Kangnam Sacred Heart Hospital (HKS IRB 2025-10-023), which waived the requirement for informed consent due to the retrospective nature of the study. The patient selection process is summarized in Figure 1.

Data collection and variables

**Figure 2:**
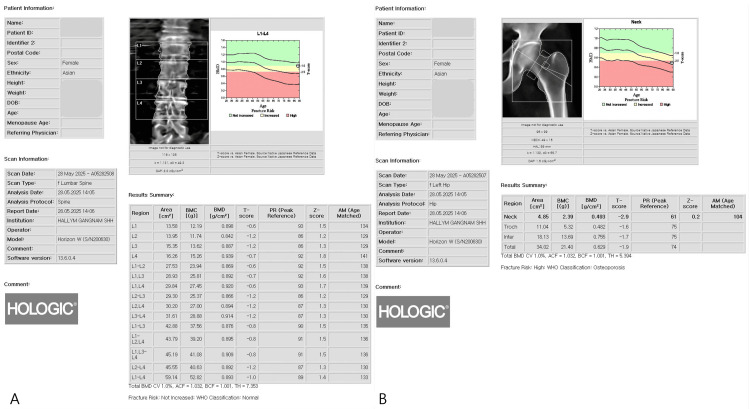
DXA assessment of BMD (A) Lumbar spine measurements showing BMD, BMC, and T-scores at each vertebral level. (B) Hip measurements demonstrating region-specific BMD, BMC, and T-scores DXA: dual-energy X-ray absorptiometry; BMD: bone mineral density; BMC: bone mineral content

**Figure 3:**
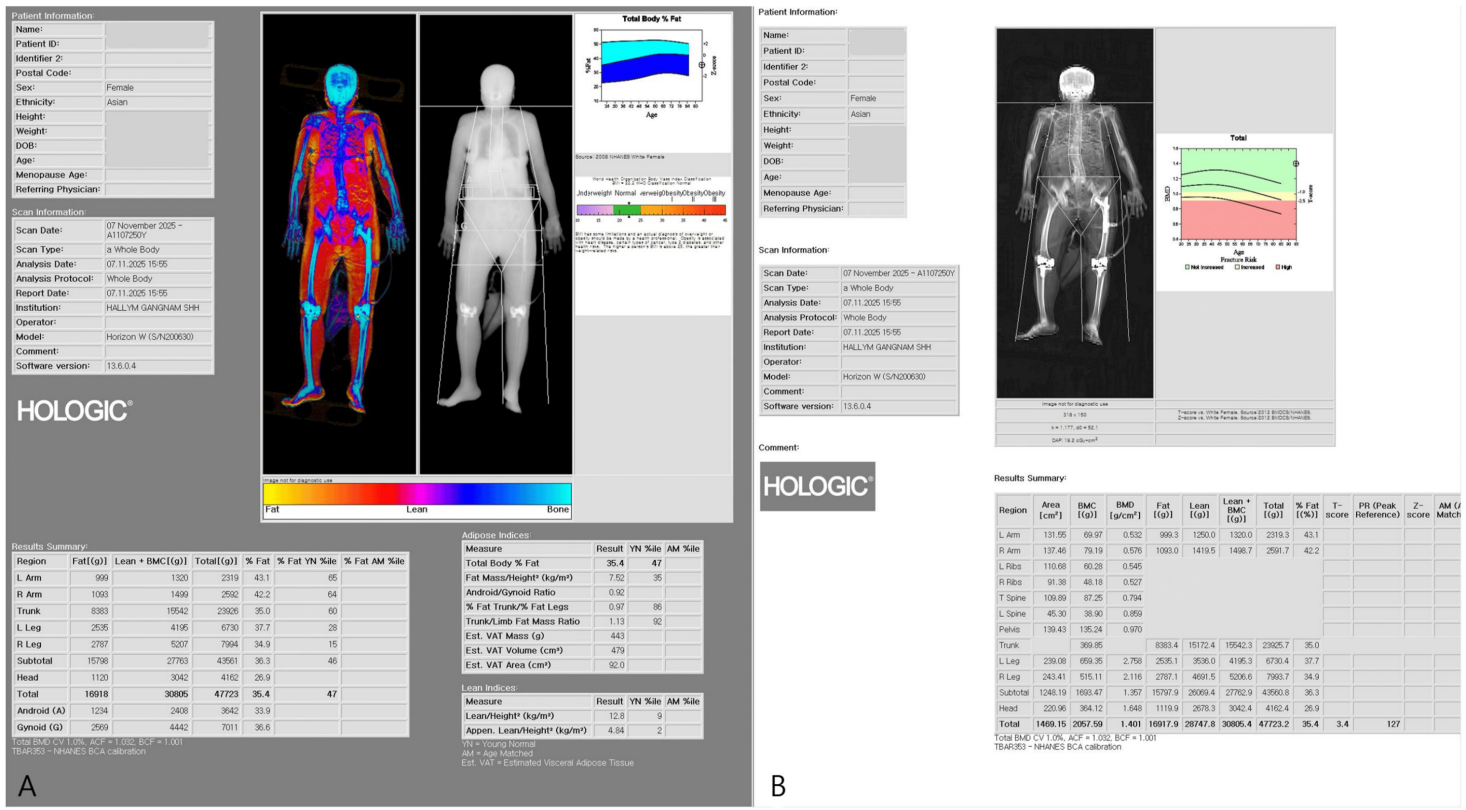
DXA-based body composition analysis (A) Global body composition indices, including ASMI, SMI, and body fat percentage. (B) Regional measurements of BMD, BMC, fat mass, lean mass, and LB for each body segment DXA: dual-energy X-ray absorptiometry; ASMI: appendicular skeletal muscle index; SMI: skeletal muscle index; BMD: bone mineral density; BMC: bone mineral content; LB: lean mass plus bone mineral content

**Figure 4:**
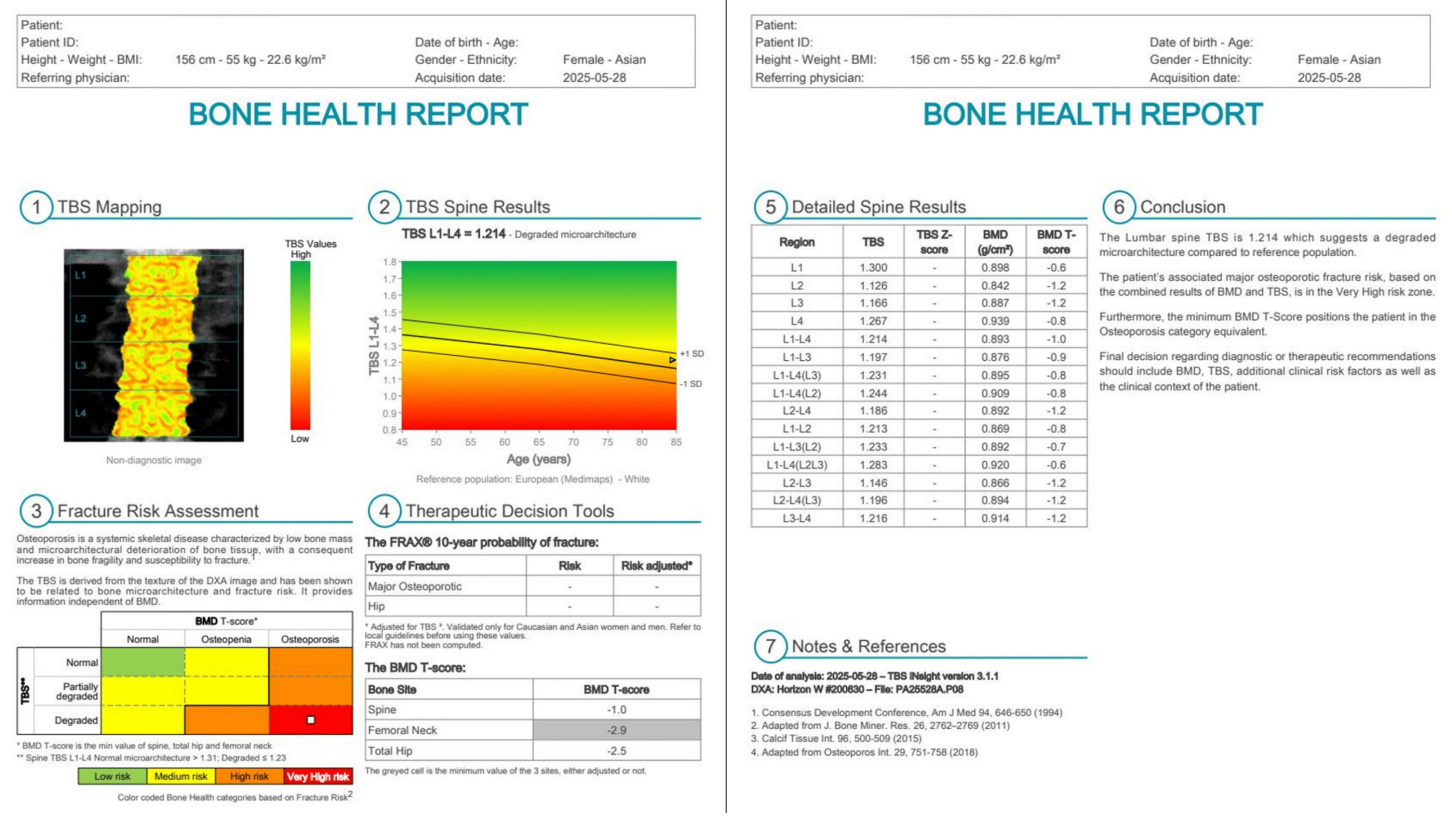
DXA-derived TBS of a representative female patient TBS was calculated from lumbar spine DXA images to assess trabecular microarchitecture independently of areal BMD DXA: dual-energy X-ray absorptiometry; TBS: trabecular bone score; BMD: bone mineral density

Whole-body and site-specific DXA were performed using a Hologic Horizon W densitometer (Hologic Inc., Marlborough, MA). All DXA examinations were conducted using standardized positioning protocols, with routine system calibration performed according to the manufacturer’s recommendations. The system software version was 13.6.0.4. Areal BMD was assessed at the lumbar spine (L1-L4) and the hip (femoral neck and total hip), and results were expressed as T-scores according to WHO criteria (Figure 2). Body composition variables included lean mass (L) and lean mass plus bone mineral content (LB) for the left and right arms and legs, as well as regional fat mass and global indices such as appendicular skeletal muscle index (ASMI) and skeletal muscle index (SMI) (Figure 3). The primary exposure variable was inter-limb asymmetry, defined as the left-right difference (Left − Right) in leg LB mass (diff_leg_LB). TBS, a DXA-derived index of lumbar spine trabecular microarchitecture that is largely independent of areal BMD, was also calculated from lumbar spine images (Figure 4). The primary outcome was fracture laterality (left or right), as documented in the medical records.

Statistical analysis

Patients were stratified into left-fracture (Fx_L, n = 71) and right-fracture (Fx_R, n = 76) groups. Continuous variables are presented as mean ± standard deviation (SD). Group comparisons for limb muscle parameters were performed using independent two-sample t-tests, and test statistic values are reported in the corresponding tables. The asymmetry indices (diff_ variables) were also compared between the two fracture groups using the same approach. A chi-square test was used to analyze the contingency between fracture side and the side with greater leg LB mass. A p-value < 0.05 was considered statistically significant. All analyses were performed to assess associations and were conducted using R software (version 4.3.1).

## Results

A total of 147 women with hip fractures were included (Fx_L, n = 71; Fx_R, n = 76), with a mean age of 79.6 ± 7.0 years. Baseline demographic and body composition characteristics, including age, height, body weight, BMI, and total body fat percentage, did not differ significantly between the fracture-side groups (Table 1).

**Table 1:** Baseline demographic and body composition characteristics by fracture side Between-group comparisons were performed using independent t-tests SD: standard deviation; BMI: body mass index

Variable	Fx_L (n = 71), mean ± SD	Fx_R (n = 76), mean ± SD	t-value	P-value
Age (years)	80.1 ± 6.9	79.1 ± 7.1	0.83	0.411
Height (cm)	153.6 ± 5.5	153.7 ± 6.3	-0.10	0.922
Body weight (kg)	54.5 ± 10.3	53.5 ± 8.3	0.64	0.526
BMI (kg/m²)	23.0 ± 3.9	22.7 ± 3.4	0.65	0.520
Total body fat (%)	38.9 ± 5.9	39.7 ± 4.7	-0.91	0.367

When muscle mass parameters were compared between groups, the left-fracture group had a significantly higher left-leg LB than the right-fracture group (p = 0.030) (Table 2). ASMI was numerically higher in the left-fracture group but did not reach statistical significance (p = 0.052), whereas SMI showed no significant difference between groups (Table 2).

**Table 2:** Comparison of limb and global muscle mass parameters between fracture-side groups Between-group comparisons were performed using independent t-tests. SD: standard deviation; ASMI: appendicular skeletal muscle index; SMI: skeletal muscle index; LB: lean mass plus bone mineral content

Variable	Fx_L (n = 71), mean ± SD	Fx_R (n = 76), mean ± SD	t-value	P-value
Lt. leg L (g)	4586.4 ± 1146.8	4290.7 ± 884.0	1.74	0.084
Rt. leg L (g)	4632.9 ± 986.7	4380.7 ± 995.8	1.54	0.125
Lt. leg LB (g)	4928.0 ± 1223.3	4539.8 ± 873.8	2.19	0.030
Rt. leg LB (g)	4867.4 ± 1030.9	4777.3 ± 1015.4	0.53	0.595
ASMI (kg/m²)	5.2 ± 0.9	4.9 ± 0.8	1.96	0.052
SMI (kg/m²)	13.3 ± 1.7	12.9 ± 1.6	11.41	0.161

Analysis of inter-limb asymmetry yielded the most striking result. The diff_leg_LB (left leg LB - Right leg LB) was positive in the left-fracture group (+60.6 g) but negative in the right-fracture group (-237.5 g), indicating that fractures tended to occur on the side with higher leg LB mass. This between-group difference was highly significant (p = 0.004) (Table 3). The magnitude of the between-group difference suggests clinically relevant asymmetry.

**Table 3:** Comparison of limb asymmetry indices between fracture-side groups Between-group comparisons were performed using independent t-tests SD: standard deviation; LB: lean mass plus bone mineral content

Variable (left - right)	Fx_L (n = 71), mean ± SD	Fx_R (n = 76), mean ± SD	t-value	P-value
diff_leg_L (g)	-46.4 ± 605.8	-90.0 ± 559.2	0.45	0.652
diff_leg_LB (g)	+60.6 ± 618.4	-237.5 ± 601.8	2.92	0.004
diff_arm_L (g)	+16.0 ± 481.2	-23.2 ± 196.5	0.64	0.524
diff_arm_F (g)	+58.0 ± 312.1	-16.2 ± 197.3	1.70	0.090

Consistent with this, a chi-square test on the contingency table (fracture side x side with higher leg LB mass) showed that 62.6% (92/147) of all fractures occurred ipsilateral to the side with greater leg LB mass (χ² = 14.1, df = 1, p = 0.0002).

## Discussion

This study presents a paradoxical finding: among older women with hip fractures, fractures occurred more frequently on the side with greater LB compared with the contralateral side. This finding challenges the intuitive assumption that the limb with lower muscle mass is inherently more vulnerable to fracture. Notably, commonly used global muscle indices such as ASMI did not differ significantly between fracture-side groups, suggesting that absolute muscle quantity alone may be insufficient to explain fracture laterality [9,10]. In contrast, inter-limb asymmetry, quantified as the left-right difference in leg LB mass (diff_leg_LB), was strongly associated with fracture laterality. Patients with left-sided fractures demonstrated relatively higher left-leg LB mass, whereas those with right-sided fractures showed relatively higher right-leg LB mass. This consistent ipsilateral pattern supports the hypothesis that asymmetry itself, rather than low absolute muscle mass, may contribute to fracture risk.

Several mechanisms may underlie this paradox. First, altered load distribution may play a role. The limb with greater LB mass may habitually bear more load during stance and gait, which could translate into higher impact forces transmitted through the ipsilateral hip during a fall [11,12]. Second, neuromuscular factors may contribute. Marked inter-limb asymmetry could impair dynamic balance and protective postural responses during a fall, potentially reducing the body’s ability to attenuate impact forces effectively, irrespective of the absolute strength of one limb [13,14]. Third, a bone-muscle mismatch may exist [6-8]. Despite higher LB mass, bone strength on that side may be insufficient relative to the forces generated or transmitted, thereby increasing susceptibility to fracture.

The clinical motivation for the present study stems from our prior work on proximal hip fractures, in which periarticular soft tissue injury was identified in over 60-70% of patients, underscoring the substantial role of non-osseous damage in hip fracture pathology [15]. In subsequent studies, we demonstrated that extracorporeal shock wave therapy could effectively alleviate postoperative pain following hip fracture surgery. However, this symptomatic improvement did not extend to reductions in mortality or major clinical endpoints [16]. These observations highlight a critical gap between pain control and overall fracture-related outcomes.

In this context, the present findings suggest that inter-limb muscle mass asymmetry may represent an upstream biomechanical factor influencing fracture laterality and load transmission at the time of injury. Even in the presence of greater muscle mass, asymmetric load bearing may expose the ipsilateral hip to higher impact forces, potentially overwhelming both bone and surrounding soft tissues. This perspective provides a plausible explanation for why interventions targeting pain or localized tissue healing alone may be insufficient to alter broader outcomes such as survival.

Limitations and strengths

This study has several limitations. First, its retrospective design and single-center setting limit causal inference and generalizability. Second, only elderly women were included, and the findings may not apply to men or younger populations. Third, data on limb dominance, muscle strength, and functional performance were unavailable, precluding evaluation of neuromuscular contributors to asymmetry. Finally, DXA-based measurements primarily reflect muscle quantity rather than muscle quality. Accordingly, these findings should be interpreted as hypothesis-generating and warrant confirmation in prospective studies incorporating functional and strength-based assessments.

Despite these limitations, our findings introduce inter-limb muscle mass asymmetry as a novel and quantifiable risk marker. From a clinical perspective, these results suggest that the assessment of bilateral symmetry, in addition to absolute muscle mass, may improve fracture risk evaluation in elderly women. Specifically, incorporating asymmetry metrics could refine prevention and rehabilitation strategies by emphasizing balanced strength and functional symmetry, potentially mitigating the risk associated with disproportionate load bearing.

The strengths of our study lend support to the credibility of this proposition. These include the use of standardized DXA protocols for body composition assessment, a well-defined clinical cohort of elderly women with low-energy hip fractures, and the novel evaluation of inter-limb asymmetry, a previously overlooked biomechanical factor.

## Conclusions

In older women with hip fractures, fracture laterality was significantly associated with the side showing greater LB. These findings suggest that inter-limb asymmetry, rather than low absolute muscle mass alone, may be a relevant biomechanical factor in hip fracture risk assessment. Prospective studies that incorporate functional measures are needed to confirm this relationship, clarify underlying mechanisms, and determine whether interventions targeting muscle asymmetry can reduce fracture risk.
